# New Appliances and Things Medical

**Published:** 1908-10-31

**Authors:** 


### NEW APPLIANCES AND THINGS MEDICAL.

[We shall be glad to receive at our Office, 23 & 29 Southampton Street, Strand, London, W.C., from the manufacturers, specimens of all new preparations and appliances which may be brought out from time to time.]

### MEDICAL PREPARATION OF MUSTARD.

Messrs. J. and J. Colman, Limited, the well-known manufacturers of mustard, of Norwich, and 108 Cannon Street, London, E.C., have sent us various samples of their preparations.

*Colman's Mustard Bran* is a convenient and satisfactory means of preparing a mustard poultice. It takes up and retains considerably more boiling water than is absorbed by linseed meal. It is cleanly in use, it does not turn rancid or quickly lose its heat, and it is light in weight. Poultices can be made very rapidly with this preparation. We observe that a muslin bag is enclosed with each package.

*Colman's Sinapisms.*—These mustard plasters are reliable rubefacients and counter-irritants. The mustard is spread on a thin pliable fabric, and is free from unpleasant smell. The action of these plasters is rapid and otherwise satisfactory. They absorb moisture very readily, and accommodate themselves well to the region of application.

*Colman's Concentrated Mustard Oil* is the natural product of the mustard-seed, and is free from admixture with ether, turpentine, or organic acids. It can be recommended as a reliable domestic embrocation.

*Colman's Mustard* is the best means for preparing the hot mustard-bath, whose value as a restorative in dangerous conditions of infantile collapse is sufficiently well known to the medical profession.

### AUSTRALIAN BURGUNDY.

(Godfree Felton and Co., Adelphi House, 71 and 72 Strand, W.C.)

We have received a sample of the above wine and have examined and used it. Before describing its individual properties we would emphasise the fact that Burgundy is an extremely useful wine from the medical standpoint. It stands in a sense mid-way between the lighter claret and the heavier port and Madeira. Many patients whose nutrition is temporarily at a low ebb, and whose digestions or diatheses will not permit the use of the stronger wines, find a most helpful and useful tonic in good Burgundy. In prescribing Burgundy—as, indeed, every other wine—the medical practitioner should accurately define both the dose and the method of taking it. A wine like Burgundy should be given undiluted and in doses of about four fluid ounces or one claret glassful. Mineral water should not be added to it, but in patients suffering from hyperacidity it may be advisable to direct them to take in a separate glass an equal quantity of some not too gaseous alkaline water. The wine is best drunk at the beginning of the meal, as it increases appetite. The Burgundy before us is of average alcoholic strength and acidity, good taste and colour, and sufficient body; in fact, we think it is in every way a sound wine and to be recommended.

